# The Role of microRNAs in Lung Cancer: Mechanisms, Diagnostics and Therapeutic Potential

**DOI:** 10.3390/ijms26083736

**Published:** 2025-04-15

**Authors:** Elżbieta Bartoszewska, Piotr Misiąg, Melania Czapla, Katarzyna Rakoczy, Paulina Tomecka, Michał Filipski, Elżbieta Wawrzyniak-Dzierżek, Anna Choromańska

**Affiliations:** 1 Faculty of Medicine, Wroclaw Medical University, Mikulicza-Radeckiego 5, 50-345 Wroclaw, Poland; elzbieta.bartoszewska@student.umw.edu.pl (E.B.); piotr.misiag@student.umw.edu.pl (P.M.); melania.czapla@student.umw.edu.pl (M.C.); katarzyna.rakoczy@student.umw.edu.pl (K.R.); paulina.tomecka@student.umw.edu.pl (P.T.); michal.filipski@student.umw.edu.pl (M.F.); 2 Student Research Group No. K148, Faculty of Pharmacy, Wroclaw Medical University, Borowska 211A, 50-556 Wroclaw, Poland; 3 Department and Clinic of Bone Marrow Transplantation, Oncology and Pediatric Hematology, Borowska 213, 50-556 Wroclaw, Poland; elzbieta.wawrzyniak-dzierzek@umw.edu.pl; 4 Department of Molecular and Cellular Biology, Wroclaw Medical University, Borowska 211A, 50-556 Wroclaw, Poland

**Keywords:** microRNAs, lung cancer, liquid biopsy, diagnostic biomarkers, prognostic biomarkers, targeted therapy, personalized medicine

## Abstract

MicroRNAs (miRNAs) are small RNA molecules that do not have coding functions but play essential roles in various biological processes. In lung cancer, miRNAs affect the processes of tumor initiation, progression, metastasis, and resistance to treatment by regulating gene expression. Tumor-suppressive miRNAs inhibit oncogenic pathways, while oncogenic miRNAs, known as oncomiRs, promote malignant transformation and tumor growth. These dual roles position miRNAs as critical players in lung cancer biology. Studies in recent years have shown the significant potential of miRNAs as both prognostic and diagnostic biomarkers. Circulating miRNAs in plasma or sputum demonstrate specificity and sensitivity in detecting early-stage lung cancer. Liquid biopsy-based miRNA panels distinguish malignant from benign lesions, and specific miRNA expression patterns correlate with disease progression, response to treatment, and overall survival. Therapeutically, miRNAs hold promise for targeted interventions. Strategies such as miRNA replacement therapy using mimics for tumor-suppressive miRNAs and inhibition of oncomiRs with antagomiRs or miRNA sponges have shown preclinical success. Key miRNAs, including the let-7 family, miR-34a, and miR-21, are under investigation for their therapeutic potential. It should be emphasized that delivery difficulties, side effects, and limited stability of therapeutic miRNA molecules remain obstacles to their clinical use. This article examines the roles of miRNAs in lung cancer by indicating their mechanisms of action, diagnostic significance, and therapeutic potential. By addressing current limitations, miRNA-based approaches could revolutionize lung cancer management, offering precise, personalized, and minimally invasive solutions for diagnosis and treatment.

## 1. Introduction

### 1.1. Overview of Lung Cancer

Lung cancer is among the most prevalent malignant tumors [1]. This disease is the final phase of multistage carcinogenesis, characterized by a progressive genetic and epigenetic alteration rather than a rapid shift from bronchia epithelioma [2]. Lung neoplasms represent diverse heterogenic forms with distinct histologies and molecular profiles. They are divided into two types, based on histology: non-small cell lung carcinoma (NSCLC) and small cell lung carcinoma (SCLC). NSCLCs are typically subdivided into adenocarcinoma, squamous cell carcinoma, and large-cell carcinoma. Both NSCLC and SCLC have extremely poor survival rates [3].

It is one of the most significant global health concerns due to its morbidity and fatality rate [4]. This disease is the primary cause of cancer-related fatalities globally, with the greatest mortality rates for both women and men [5]. SCLC accounts for 10–15% of all lung cancers, while NSCLC is diagnosed in 80–85% of cases. The average age at which a patient’s lung cancer is determined is around 70 years old [6]. The 5-year survival rates range from 4% to 17% based on the extent of the illness at diagnosis [2]. There is a great number of risk factors, with tobacco smoking being the primary one, accounting for 80–90% of new incidents [7].

Despite advancements in non-invasive testing, only 10–15% of lung cancer cases are discovered at an early stage. Seventy-five percent of patients with lung cancer are detected at an advanced stage, with few treatment choices available. Current diagnostic tools, such as sputum cytology and chest radiography, are insufficient for diagnosing NSCLC. Additionally, tumor markers like CEA (carcinoembryonic antigen), NSE (neuron-specific enolase), CYFRA 21-1 (a soluble fragment of cytokeratin 19 associated with epithelial cell tumors), and SCCA (squamous cell carcinoma antigen) do not allow for early detection of lung cancer [2].

Circulating microRNAs (miRNAs), which reflect tumor/host exchanges, have been identified as possible biomarkers for cancer detection and prognosis, regardless of tumor stage or mutational burden. The studies examined the role of miRNA-based liquid biopsies in the context of Low-Dose Computed Tomography (LDCT) screening. The miRNA signature classifier (MSC) and the miR-Test reduced the LDCT-false positive rate by five- and four-fold while maintaining comparable specificity (75–81%) and sensitivity (78–87%). In post-surgical plasma samples, the MSC performed well in monitoring disease recurrence [8].

### 1.2. Introduction to microRNAs (miRNAs)

**Figure 1:**
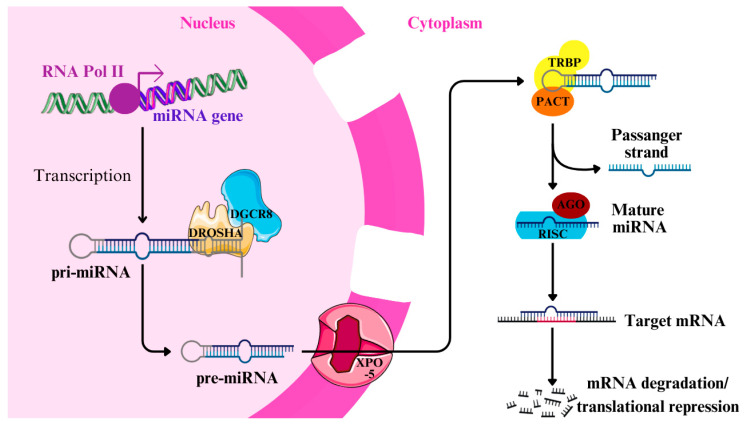
Biogenesis of miRNA. In the nucleus, primary miRNA is generated via RNA polymerase II. Afterward, Drosha and DGCR8 trim pri-miRNA, resulting in the formation of pre-miRNA, which is exported by Exportin-5 to the cytoplasm. There, the Dicer complex, consisting of TAR RNA-binding protein (TRBP) and protein activator of interferon-induced protein kinase (PACT), regulates miRNA maturation. Finally, the RNA-induced silencing complex (RISC) complex is created and binds to Argonaute (AGO) protein. Mature RISC complex attaches to target mRNA at complementary locations, causing translational inhibition or target destruction [15].

RNA molecules perform critical and various functions in many biological processes. Some of them are carried out by certain forms of non-coding RNA, known as small non-coding RNAs (sncRNA), made of fewer than 200 nucleotides. One of the most important types of sncRNA is microRNA (miRNAs) (Table 1) [9]. miRNAs are short, non-coding transcripts of RNA (19–25 ribonucleotides), which are not translated into peptides but can regulate gene expression post-transcriptionally [10]. Genes that encode miRNAs are found in protein-coding genes’ introns or exons and in intergenic regions, which are frequently unstable (Figure 1) [11].

They are generated via two pathways: the canonical and non-canonical. In the canonical pathway, the genes are transcribed by RNA polymerase II to form a primary transcript, called the pri-miRNAs, from intronic regions. The pri-miRNAs feature a hairpin or stem-loop structure consisting of an upper and lower stem, a terminal loop, and flanking single-stranded sequences. They are then processed by a microprocessor complex composed of Drosha and DGCR8, which trims them into pre-miRNAs (~60–70 nucleotides). The new molecule retains the stem/loop structure, which is crucial for recognition by the export machinery. These precursor miRNAs are exported from the nucleus to the cytoplasm, mostly by Exportin-5 (XPO-5) [12,13].

In the cytoplasm, the Dicer enzyme, made of a protein activator of the interferon-induced protein kinase (PACT) and TAR RNA-binding protein (TRBP), cleaves the pre-miRNA into a mature form. The precision of cleavage by Drosha and, subsequently, Dicer is influenced by the terminal loop and the length of the stem of pre-miRNA. The resulting miRNA is created by cleaving off the terminal loop, producing a double-stranded miRNA/miRNA* duplex (passenger strand). One strand, the mature miRNA, is incorporated into the RNA-induced silencing complex (RISC) with an Argonaute (AGO) protein, where it functions in gene regulation, while the passenger strand is typically degraded. The mature miRNA has a characteristic length of ~22 nucleotides and contains a seed sequence (positions 2–8) critical for target recognition. In the alternative route, miRNAs are derived directly from introns, bypassing the need for Drosha processing. The subsequent steps of maturation mirror those in the canonical pathway [12,13].

In the cytoplasm, miRNAs bind to the coding regions and 3′ and 5′ untranslated regions (UTRs) of messenger RNA (mRNA), causing degradation or temporary translation suppression [14]. miRNAs can degrade mRNA by recruiting deadenylation complexes like PAN2/3, CCR4, and the NOT complex, which facilitate degradation through 5′-3′ exonucleases. They can also inhibit translation by interacting with eukaryotic initiation factors without affecting mRNA stability [12]. Downregulation of a specific miRNA causes overexpression of the relevant protein’s expression. miRNA upregulation results in reduced expression of the target protein. miRNAs also bind to the gene’s promoter region, inducing gene transcription [14]. For example, transfecting miR-24-1 has been observed to enhance the expression of nearby genes. Furthermore, the regulatory activity of miRNAs can be modulated by competing endogenous RNAs (ceRNAs), such as circRNAs, pseudogenes, and lncRNAs. These ceRNAs bind to miRNAs, preventing them from interacting with their target mRNAs, thereby blocking their regulatory effect [12].

A single molecule of miRNA can affect thousands of genes and around 30% of mRNAs [11]. Furthermore, miRNA seems to influence the targeted gene expression extremely specifically. This allows for precise control of physiological responses [10]. Endogenous miRNAs influence cell functions such as DNA repair, proliferation, cell differentiation, apoptosis, and metabolism. The biological role of circulating miRNAs is yet to be studied. Depending on the genes they control, miRNAs can operate as oncomirs (procancerogenic) or suppressor miRNAs (block oncogenes). It is hypothesized that certain extracellular miRNAs can transport information between cells through a variety of normal and pathological pathways [11].

It has been proposed that a certain miRNA pattern (decrease or increase in expression) in blood and tissue may be typical to a certain disease, allowing for the use of chosen miRNAs to monitor patients’ conditions [11]. The studies have shown many correlations between changed miRNA levels and the physiopathology of a variety of processes, including cardiovascular, mitochondrial, inflammatory, immune disease, neurodegenerative, rare genetic conditions, and others [13]. The detection of miRNAs in different bodily fluids, such as blood, urine, saliva, milk, bronchial lavage, cerebrospinal fluid, and synovial fluid, has considerably increased their application in diagnostics [11]. The focus of most of these studies has been on miRNAs as cancer biomarkers, highlighting their importance as individualized theranostic factors. Moreover, miRNAs have the ability to serve not only as predictive markers at the beginning and course of neoplastic illness but can also be used to analyze therapeutic response and drug resistance [13].

This review aims to explore the potential of miRNAs as a diagnostic and prognostic biomarker for lung cancer, focusing on their role in improving early detection and monitoring disease progression. This review will analyze the mechanisms of tumor suppressors and oncogenic miRNAs and consider their roles as potential signature molecules and therapeutic targets.

**Table 1:** Biogenesis of miRNAs [11,12,13,14,15].

Origins of miRNAs	—Introns and Exons: miRNAs can originate from introns and noncoding exons. —50% Noncoding: About 50% come from non-protein-coding transcripts, regulated by their promoters. —Intragenic miRNAs: Derived from intragenic regions, mostly introns. —Clusters: Some miRNAs are clustered and expressed as polycistronic transcripts.
Examples of miRNA Clusters	—miR-17/92 Cluster: Contains miR-17, miR-18, miR-19, miR-19b, miR-20a, and miR-92a, located on chromosome 13. —Paralogues: miR-106b/25 on chromosome 7 and miR-106a/363 on chromosome X.
Canonical vs. Noncanonical Pathway	—Canonical: Involves Drosha, Dicer, and XPO-5. —Noncanonical: Includes mirtrons (spliced introns) and m7G-capped pre-miRNAs, bypassing Drosha/DGCR8 or Dicer.
Biogenesis Pathway	1. Transcription: RNA polymerase II transcribes pri-miRNA. 2. Processing in Nucleus: Microprocessor complex (Drosha + DGCR8) cleaves pri-miRNA into pre-miRNA. 3. Export: Exportin 5 (XPO-5) transports pre-miRNA to the cytoplasm. 4. Processing in Cytoplasm: Dicer processes pre-miRNA into mature miRNA.
Role of Proteins in Biogenesis	—Drosha: Cleaves pri-miRNA. —Dicer: Processes pre-miRNA to mature miRNA. —XPO-5: Transports pre-miRNA. —RISC: RNA-induced silencing complex involving Argonaute (AGO1-4) proteins.
Functions in RISC	—Guide Strand (-5p): Loaded into RISC, binds target mRNA. —Passenger Strand (-3p): Usually degraded, though some have functions. —Mechanism: Targets mRNAs for degradation or translational repression.
Determinants of Function	—Complementarity: Full complementarity leads to mRNA degradation; partial complementarity results in translational repression. —Cell State: Influences miRNA abundance and target mRNA availability.
Dual Roles in Regulation	—Repression: Inhibits gene expression in the cytoplasm. —Activation (RNAa): Some miRNAs activate gene expression in the nucleus by recruiting transcription factors (e.g., miR-24-1).
Biological Processes	—Development: Nervous system development, differentiation (e.g., let-7 and miR-290-295). —Cancer: p53 regulation (miR-34, miR-145, etc.). —Mitochondrial Function: MitomiRs regulate oxidative phosphorylation (e.g., miR-378).

## 2. Mechanisms of miRNA Involvement in Lung Cancer

### 2.1. Tumor Suppressor miRNAs

MicroRNAs (miRNAs) are pivotal regulators of gene expression, playing a significant role in the initiation and progression of cancer. They are generally classified into tumor suppressor miRNAs (TSmiRs), which inhibit tumor growth, and oncomiRs, which promote cancer development. A single miRNA can regulate over 200 target genes, including transcription factors, receptors, and transporters [16]. The extensive interactions of miRNAs complicate the identification of specific transcripts and pathways that they regulate [17]. Furthermore, the regulation of tumor-suppressor miRNA expression extends beyond cancer cells to the tumor microenvironment (TME) [18].

Mechanisms through which miRNAs function as tumor suppressors include mRNA cleavage or repression, inhibition of translation (e.g., blocking cap recognition or preventing ribosome 60S subunit attachment), ribosome detachment, premature translation termination, and co-translational protein degradation [19]. Typically, miRNAs interact with the 3′ untranslated region (3′ UTR) of target mRNAs to induce mRNA degradation and translational repression [20].

The miR-200 family is recognized for its role as a tumor suppressor in epithelial-to-mesenchymal transition (EMT), a process that is essential for cancer metastasis. miR-200 targets ZEB1 and ZEB2, transcriptional repressors of E-cadherin. Upregulation of miR-200 enhances E-cadherin expression, thereby reducing cell mobility and inhibiting cancer cell invasion and metastasis [21]. miR-204-5p induces apoptosis in lung cancer cells by downregulating BCL2, an anti-apoptotic protein. The reduced expression of BCL2 facilitates the release of cytochrome C from the mitochondria, activating caspases 3 and 7, which, in turn, leads to apoptosis and a reduction in cell viability [22].

miR-126 exhibits cell-dependent functions and plays distinct roles in regulating angiogenesis in endothelial cells (ECs) and tumor angiogenesis. In ECs, miR-126 promotes angiogenesis by stimulating cell proliferation and migration, suppressing negative regulators like Spred-1 and PIK3R2, and inhibiting Dlk1. However, in tumors such as lung cancer, miR-126 can inhibit tumor angiogenesis by lowering VEGFA levels and activating signaling pathways such as Akt/mTOR/Erk1/2, ultimately suppressing tumor growth [23].

miR-15a-5p inhibits the proliferation, migration, and invasion of lung cancer cells by modulating lipid metabolism [24]. It disrupts fatty acid synthesis by inhibiting acetate uptake and reducing acetyl-CoA activity, which in turn decreases H4 histone acetylation through the suppression of ACSS2 expression. This metabolic alteration limits the cellular energy supply necessary for tumor cell growth and migration. Additionally, miR-15a-5p is transported to the nucleus under hypoxic conditions, where it directly inhibits ACSS2 function, reducing acetylation of histones and suppressing cancer metastasis [25].

The let-7 miRNA family plays a pivotal role in regulating multiple oncogenes, including RAS, MYC, HMGA2, cyclin-dependent kinase 6 (CDK6), and cell division cycle 25A (CDC25A) [26,27,28]. Kumar et al. demonstrated that members of the let-7 family inhibit NSCLC proliferation by directly targeting K-Ras and cyclin D1 [29]. This inhibitory effect has been demonstrated both in vitro and in vivo, where let-7 effectively reduces tumor growth in mouse models of lung cancer [21].

Recent studies have demonstrated that the ectopic expression of miR-34b-3p, which is downregulated in non-small cell lung cancer (NSCLC) tissues and cell lines such as A549 and H1299, inhibits cell proliferation and cell cycle progression and induces apoptosis by targeting CDK4, a key regulator of G1 phase progression [30]. Detailed mechanisms and targets of individual tumor-suppressor miRNAs can be found in Table 2.

### 2.2. Oncogenic miRNAs (oncomiRs)

Oncogenic miRNAs (oncomiRs) constitute numerous functional subgroups of microRNAs and perform their roles through various molecular mechanisms (Table 3). For instance, in non-small-cell lung cancer (NSCLC), miR-411-5p and miR-411-3p have been reported to foster the oncogenic expansion of both cell lines and tissues, as they promote cancer cell proliferation and migration as well as inhibit apoptosis. The molecular mechanism that underlies their pro-neoplastic characteristics is based on targeting sprouty RTK signaling antagonist 4 (*SPRY4*) and, subsequently, activating epidermal growth factor receptor (EGFR) and protein kinase B (AKT) signaling, as well as epithelial/mesenchymal transition (EMT). What is more, miR-411-5p also reduces molecular levels of thioredoxin interacting protein (*TXNIP*), a tumor suppressor that regulates cell cycle progression [46].

**Figure 2:**
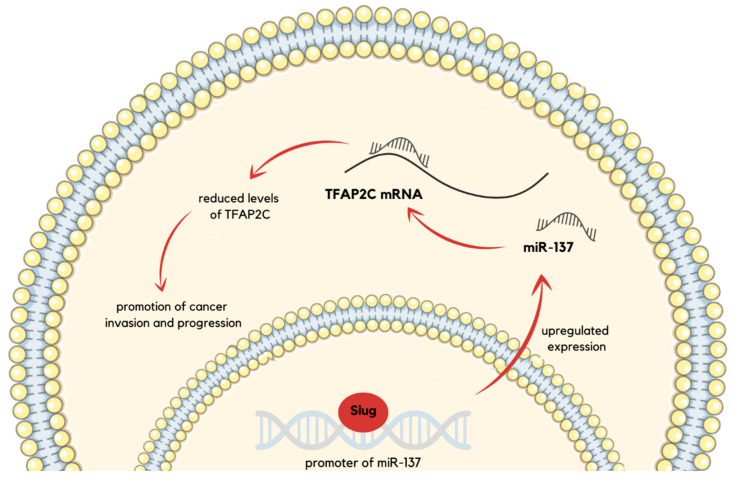
Schematic visualization of the oncogenic activity of miR-137 in lung cancer progression. miR-137 is transcriptionally activated by Slug, which binds to the E-box in its promoter. Once expressed, miR-137 suppresses the *TFAP2C*, leading to increased cancer cell invasion and progression. Clinical data support this mechanism, as low Slug and miR-137 expression, along with high *TFAP2C* levels, correlate with better survival rates in lung adenocarcinoma patients [47].

miR-137 constitutes another important oncomiR downstream of Slug (the protein product of SNAI2), a transcription factor that functions as an EMT regulator. Slug binds to miR-137 promoter E-box and upregulates its expression, which promotes cancer invasion and progression through transcription factor AP-2 gamma (*TFAP2C*) suppression (Figure 2). Clinical data supports those molecular findings, as in patients diagnosed with lung adenocarcinoma, low Slug and miR-137 expression and high *TFAP2C* expression are associated with better survival rates [47]. Other significant oncomiRs, miR-17, miR-18a, miR-19a, miR-20a, miR-19b-1, and miR-92-1, constitute oncogenes that form miR-17-29 cluster residing in 13q31.3 [48]. Enumerated members of the cluster are overexpressed in small-cell lung cancer (SCLC) [49]. Functionally, they cooperate with c-Myc, which results in the promotion of tumor development and neovascularization [50].

The most recent studies suggest that particular types of oncomiRs may be connected with specific lung cancer metastases, which lets us perceive them as prospective biomarkers or therapy targets. miR-21 is overexpressed in lung cancer patients with brain metastasis. Increased miR-21 expression activates ERK-STAT3 signaling pathways via inhibiting diacylglycerol kinase (DGKB) and thus promotes metastasis. Targeting the miR-21/ERK/STAT3 pathway with ulixertinib reduces brain metastasis incidence and increases cancer survival in a mouse model [51].

**Table 3:** List of oncomiRs upregulated in lung cancer and their functions.

OncomiRs	Targets/Regulators	Function	Ref.
**OncomiRs Related to Proliferation and Growth**			
miR-31	*LATS2*, *PPP2R2A*	Through repressing tumor suppressors, *LATS2* and *PPP2R2A*, miR-31 promotes cancer growth.	[52]
miR-411-5p, miR-411-3p	*SPRY4*, *TXNIP*	Targeting suppressor genes *SPRY4* and *TXNIP* results in carcinogenesis promotion.	[46]
miR-1290, miR-1246	P53, *THBS2*	miR-1246 and miR-1290 inhibition decreases stemness markers and EMT markers, and thus, anti-miR-1246 and anti-miR-1290 suppress proliferation and invasion of NSCLC.	[53]
miR-211	*SRCIN1*	Downregulating *SRCIN1* expression by miR-211 promotes NSCLC proliferation.	[54]
miR-196a	*FoxO1*, *p27*, *HOXA9*	NSCLC proliferation and migration are stimulated through direct *FoxO1*, p27, and HOXA9 targeting.	[55]
miR-324-5p, miR-324-3p		miR-324-5p promotes both cell proliferation and invasion in lung cancer cells. miR-324-3p significantly increases cell proliferation but does not alter the invasive profile of cancer cells.	[56]
miR-19	*CBX7*	miR-19 plays the role of a tumor accelerator, as it promotes lung cancer cell proliferation by inhibiting the expression of *CBX7*.	[57]
miR-1269a	*SOX6*	Through downregulating *SOX6* expression, miR-1269a promotes NSCLC growth.	[58]
miR-17, miR-18a, miR-19a, miR-20a, miR-19b-1, miR-92-1		In cooperation with the c-Myc miR-17-29 cluster promotes tumor development and neovascularization. miR-20a may regulate genes associated with TGF-β and VEGF.	[50,59]
OncomiRs involved in metastasis and invasion			
miR-137	*TFAP2C*	Cancer invasion and progression are promoted through *TFAP2C* suppression.	[47]
miR-490-3p	*PCBP1*	Targeting *PCBP1* regulates cancer metastasis.	[60]
miR-451a		Increased level of miR-451a is associated with lymph node metastasis and vascular invasion.	[61]
miR-574-5p	*PTPRU*	miR-574-5p is overexpressed in patients with advanced metastatic NSCLC. It promotes both the migration and the invasion of cancer cells as well as enhances the tyrosine phosphorylation of β-catenin by repressing *PTPRU* expression in vitro.	[62]
miR-21, miR-155	*SOCS1*, *SOCS6*, *PTEN*; *DGKB*	miR-21 and miR-155 promote the development of NSCLC by downregulating *SOCS1*, *SOCS6*, and *PTEN*. miR-21 also promotes metastasis to the brain through the *ERK-STAT3* signaling pathway, which inhibits *DGKB*.	[51,63]
OncomiRs connected with immune modulation			
miR-320a	*STAT4*	mir-320a secreted by neutrophils of high-risk heavy smokers promotes the M2-like immunosuppressive phenotype of macrophages through *STAT4* downregulation.	[64]
OncomiRs related to apoptosis and therapy resistance			
miR-208a	p21	miR-208a increases the proliferation of lung cancer cells and decreases cellular apoptosis. What is more, it enhances the radioresistance of the A549 lung cancer cells.	[65]
miR-146a	*CHOP*	Though downregulating *CHOP* expression, miR-146a induces chemotherapy resistance in lung cancer.	[66]
Prognostic markers			
miR-23b-3p, miR-10b-5p, miR-21–5p		Elevated levels of miR-23b-3p, miR-10b-5p, and miR-21-5p are independently associated with poor overall survival in NSLCC patients. miR-21-5p might be involved in tumor progression; however, its exact pathological mechanism needs to be further examined.	[67]

## 3. miRNAs as Biomarkers in Lung Cancer Diagnostics

### 3.1. miRNA Detection Methods

miRNAs may be detected using several methods. Quantitive Reverse Transcription Polymerase Chain Reaction (qRT-PCR) is the most widely used for miRNA analysis due to its high specificity and sensitivity. However, qRT-PCR is predominantly designed for the quantification of known miRNAs and lacks the capability for de novo identification of novel miRNA sequences [28]. miRNAs microarray allows to detection of genes and miRNAs with differential expression. Subsequently, the interactions between differently expressed genes and miRNAs may be predicted using bioinformatics methods. These methods include functional enrichment analysis, miRNA/gene regulatory networks, protein/protein interaction (PPI) networks, and competing endogenous RNA (ceRNA) networks [68]. Next Generation Sequencing (NGS) is also commonly used in lung cancer miRNA detection [69]. Fluorescence In Situ Hybridization (FISH) is used in this approach. Moreover, FISH allows to assay multiple targets simultaneously and displays them with a single specimen [70]. Another method is electrochemical or optical biosensors, which can detect low concentrations of targeted miRNA [71].

Recent studies have focused on standardizing pre-analytical and analytical workflows for the clinical use of circulating free miRNAs (cfmiRNAs) in cancer patients, including the evaluation of extraction protocols and benchmarking of quantification platforms. However, clinical implementation remains limited due to challenges such as pre-analytical variability, extraction and quantification biases, and the absence of universally accepted reference genes for normalization. Missing consensus of the used matrix and unsatisfactory storage conditions are significant in pre-analytical variability [72]. Currently, a primary difficulty involves selecting the most suitable normalization method. miRNA-16 is often employed for normalization despite multiple studies reporting that it is deregulated in cancer patients’ plasma samples and is susceptible to hemolysis [73,74].

The combination of biomarkers demonstrated superior diagnostic accuracy for detecting NSCLC compared to individual miRNAs. For example, miRNA panels (Table 4) incorporating miRNA-210 exhibited the aforementioned characteristics more effectively than those using miRNA-210 alone. Specifically, it showed higher sensitivity (0.76 vs. 0.66), specificity (0.88 vs. 0.79), positive likelihood ratio (6.5 vs. 3.2), and diagnostic odds ratio (23 vs. 7), along with a lower negative likelihood ratio (0.27 vs. 0.43) and a greater area under the curve (0.91 vs. 0.80) [75].

#### Novel Technologies for microRNA Detection and Analysis

Various methods have been utilized for miRNA detection, including Northern blot analysis, qRT-PCR, RNA sequencing, nanopore sensing, and microarray [84,85,86,87,88]. Of these, Northern blotting is considered the gold-standard miRNA assay, yet it necessitates substantial DNA sample volumes, employs radiolabeled probes, and involves time-consuming and labor-intensive protocols, in addition to having low sensitivity—factors that restrict its broader application in clinical settings [89]. Currently, qRT-PCR is the most frequently employed miRNA assay owing to its high sensitivity and wide dynamic range [90]. However, it is hindered by complex probe design and the need for precise temperature cycling control [91]. While RNA sequencing enables both the sequencing and quantification of miRNA, it demands extensive and time-consuming data analysis as well as significant financial investment [92].

Nanopore-based miRNA biosensors offer specificity at the single-molecule scale without the need for either labeling or amplification [93]. Nonetheless, unwanted interactions with nontarget nucleic acids within the nanopore inevitably compromise detection accuracy [94]. Microarray-based approaches can detect multiple miRNAs simultaneously because of their high-throughput screening capability; however, they still require expensive instrumentation, intricate probes, and well-trained personnel [95]. Consequently, there is a strong motivation to develop novel biosensors with enhanced sensitivity, straightforward operation, and improved user-friendliness.

CRISPR/Cas technology has been utilized for miRNA biosensors. Building on the RNA-sensing ability of Cas13a, a recent study combined the trans-cleavage activity of CRISPR/Cas13a with an electrochemical enzymatic readout within a microfluidic biosensing platform, enabling the rapid detection of microRNAs. This biosensing system demonstrated the ability to detect miRNA-19b at picomolar concentrations in both buffered solutions and human serum samples, with a total analysis time of under 4 h [96]. The alternative approach of using biosensors involves using nanoparticle-based and enzymatic amplification to improve sensitivity [97]. For instance, Moazampour et al. have created a label-free electrochemical biosensor using ZnS quantum dots functionalized with L-cysteine to detect miR-200a. It demonstrated remarkable sensitivity, achieving a detection limit of 8.4 fM, and effectively distinguished closely related miRNA sequences, thus highlighting its selectivity [98].

Electrochemiluminescence (ECL) biosensors may be used for miRNA detection. Annihilation-based ECL, co-reactant-based ECL, and co-reaction accelerator-based ECL are the three main approaches [99]. Crucially, the co-reactants remain unreactive toward the substrates and analytes in the reaction mixture, leading to minimal interference in both the analytical process and its outcomes. Consequently, the pronounced signal intensity and low background noise inherent to co-reactant-based ECL serve to enhance miRNA detection performance [100,101]. Thanks to the synergistic interplay of co-reaction accelerators and O₂ as a co-reactant, the co-reaction accelerator-based ECL demonstrates a notably amplified signal, indicating strong potential for miRNA detection [102]. Annihilation-based ECL generally involves luminophores with extended excited-state lifetimes and electrochemically produced species that can interact with these luminophores. Owing to its high sensitivity and selectivity, annihilation-based ECL has been extensively utilized for miRNA detection [103,104].

Artificial intelligence (AI) is an emerging tool that has already been proven successful in a number of medical fields [105]. One of the most useful aspects of AI is machine learning (ML), which automatically enables a machine or system to learn and improve from the already existing data [106]. MicroRNA is a great biomarker for AI-based models focusing on the subtyping of cancer in its early stages and its prognosis, particularly through the integration of microfluidics technology. This innovation allows for diagnosis confirmation based on a minimal sample volume. Moreover, AI algorithms are not only able to detect subtle variations in expression levels but can also accelerate the interpretation of complex sets of data. This is a necessary step to take to advance personalized medicine [107]. MicroRNA-based ML models are already being used in the detection and analysis of not only lung cancer but also many other neoplasms. Further development of those models and their popularization could improve diagnostic precision while substituting or limiting the usage of more intrusive diagnostic procedures, bringing comfort to patients [106].

### 3.2. Early Detection—The Potential of Circulating miRNAs in Early Lung Cancer Diagnosis

Early detection of lung cancer is associated with better outcomes and reduced mortality [76,79,108,109]. Moreover, clinically apparent symptoms occur in advanced-stage disease [76]. Blood-based assays enable the uncovering of asymptomatic disease or may facilitate diagnosis when equivocal findings from PET or CT are present [108]. Early diagnosis markers of lung cancer involve miRNAs in body fluids such as miRNAs-25, 223, 141, 155, and 1254 [110]. miRNAs-125a-5p, 25, and 126 might be potentially used in the early detection of lung cancer (Table 5). Their usefulness was proven by a 0.936 AUC value with 87.5% sensitivity and 87.5% specificity [76].

miRNAs-126, 145, 210, and 205-5p exhibit different expressions among lung cancer patients vs. cancer-free smokers. They display the best prediction of 91.5% sensitivity and 96.2% specificity in lung cancer detection based on plasma miRNA level. This study suggests that blood-based evaluation of miRNA might be used for early-stage lung cancer diagnosis [111]. Tulinsky et al. have shown that the key serum miRNA biomarkers for the early detection of lung cancer are miR-143, let-7g, miR-126, let-7a, and miR-145 [78].

Ying et al. have investigated a panel of five miRNA biomarkers that are associated with NSCLC and have potential in early-stage NSCLC diagnosis. Those miRNAs include miRNAs-7a-5p, 375, 1-3p, 1291. miRNAs-7a-5p and 375 have lower expression and miRNAs-1-3p, 1291, and 214-3p have higher expression compered to healthy individuals. Moreover, this panel displays better AUC than ctDNA-based liquid biopsy in stage I and II NSCLC. These five miRNA panels may detect stage I NSCLC with 83% sensitivity and 91% specificity. Therefore, it may be used independently for stage I NSCLC screening or combined with LDCT or other imaging methods with tissue biopsy. However, this approach requires validation in large-scale prospective studies [112].

It is worth mentioning that serum miRNA is useful among asymptomatic high-risk individuals. Bianchi et al. created the 34-miRNA model, which can detect asymptomatic NSCLC and distinguish benign and malignant lesions. Furthermore, this model has the highest AUC of 0.89 for stage I tumors, but it may detect stage II-IV tumors with an AUC of 0.88. Thus, this model can be used for all stages of NSCLC [79]. Zheng et al. found that miRNAs-155, 197, and 182 are overexpressed in the plasma of lung cancer patients, and they can be potential biomarkers. It is important to note that miRNAs-155 and 197 expression depend on metastasis. Their expression is higher in lung cancer patients when metastasis occurs [109].

Nonetheless, Arab et al. have shown that only miRNA-141 may be an auspicious biomarker for early diagnosis of NSCLC. Moreover, miRNA-21 expression is strongly positively correlated with the stage of NSCLC [113]. Serum miRNA-942 and miRNA-601 were significantly upregulated in NSCLC and outperformed traditional markers CEA, CYFRA21-1, and SCCA for early diagnosis. Combining these miRNAs further improved early-stage NSCLC detection efficacy [114]. Different expressions of miRNAs-21-5p, 181-5p, and 155-5p are associated with early-stage and late-stage male lung squamous cell carcinoma (LUCS). Thus, it might be used for male LUCS diagnosis with high accuracy [115]. miRNA-3692-3p expression is increased in NSCLC patients. However, the expression of miR-3692-3p was not associated with the treatment response or survival outcomes [116].

miRNAs-199a−3p, chr17_10932, 148a−3p, 210−3p, chr1_1402, 378d, and 138−5p display higher expression in lung adenocarcinomas (LUAD) patients. The aforementioned seven miRNAs panel demonstrates promise as a tool for the early diagnosis of LUAD presenting with GGNs [117]. Pavel et al. have shown that adding miR-146a-5p to the mRNA improves AUC from 0.66 to 0.71, which was evaluated using the ROC AUC method. This evidence has shown that miRNA combined with mRNA might enhance overall diagnostic accuracy [118]. Moreover, Abdipourbozorgbaghi et al. investigated miRNAs-210-3p and 301a-5p, which may be used as biomarkers of the early stages of LUAD and LUCS patients [119].

Pastorino et al. have shown that MSC may upgrade the overall response of screening among baseline indeterminate or positive LDCT results. Nonetheless, miRNA signature may not be used as a complement to baseline screening [120]. It is worth noting that sputum miRNA might be used for the early detection of NSCLC. However, this approach requires further research [121]. miRNA-21 expression in sputum is higher in lung cancer patients; thus, it might be used in lung cancer diagnosis. Moreover, sputum miRNA-21 detection has higher sensitivity than sputum cytology [122]. It is worth mentioning that sputum miRNA-205 is overexpressed in squamous cells in lung cancer with the best prediction. Therefore, sputum miRNA-205 estimation might be used in the diagnosis of early-stage squamous cell lung cancer with acceptable accuracy [123].

A multi-phase study revealed that miR-324-3p was significantly upregulated, while miRNA-1285 was significantly downregulated in the plasma of stage I LUCS patients compared to healthy controls with AUC at 0.79 and 0.85 for miRNA-324-3p and 1285. It is worth mentioning that their combination improved accuracy to 0.89. Thus, miRNA-324-3p may serve as an independent prognostic predictor for early-stage LUCS [124]. Moreover, miRNA combination with proteins may be useful in the early detection of lung cancer. miRNAs-320a-3p, 210-3p, 92a-3p, 21-5p, and 140-3p combination with four-protein marker panel (4MP) protein consisting of the precursor form of surfactant protein B (Pro-SFTPB), cancer antigen 125 (CA125), carcinoembryonic antigen (CEA), and cytokeratin-19 fragment (CYFRA21-1) display enhanced sensitivity at 95% specificity by 19.1% [108].

**Table 5:** The summary of miRNAs associated with early lung cancer detection alongside their characteristic features.

miRNA/miRNAs	Connection to Cancer/Cancer Type	Characteristic Features	Ref.
miRNA-223	Early detection of lung cancer.	Potential biomarker detectable in body fluids.	[110]
miRNA-141	Early detection of NSCLC.	Described as an auspicious biomarker for early NSCLC diagnosis.	[113]
miRNA-155	Early detection of lung cancer; linked to metastasis.	Overexpressed in plasma; expression increases with lung cancer metastasis.	[109,110]
miRNA-1254	Early detection of lung cancer.	Part of an early-diagnosis miRNA set detectable in body fluids.	[110]
miRNAs-125a-5p, 25, and 126	Early detection of lung cancer.	The combined panel of miRNAs-125a-5p, -25, and -126 may be potentially used in early lung cancer diagnosis.	[76]
miRNA-145, 210, and 205-5p	Early detection of lung cancer.	Differentially expressed in lung cancer vs. cancer-free smokers.	[78,111]
miRNA-143, let-7g, and let-7a	Early detection of lung cancer.	Key serum biomarker.	[78]
miRNAs-7a-5p and 375	Early-stage NSCLC.	Downregulated compared to healthy controls in NSCLC, and they are part of the same 5-miRNA panel.	[112]
miRNA-1-3p, 1291, and 214-3p	Early-stage NSCLC.	Upregulated compared to healthy controls in NSCLC, and they are part of the same 5-miRNA panel.	[112]
34-miRNAs model	NSCLC, all stages.	Detects asymptomatic NSCLC and distinguishes benign from malignant lesions with better AUC for stage I than for stages II-IV.	[79]
miRNA-197	Early detection of lung cancer; linked to metastasis.	Overexpressed in plasma; higher expression in metastatic disease.	[109]
miRNA-182	Early detection of lung cancer.	Overexpressed in plasma.	[109]
miRNA-21	NSCLC, correlation with stage also sputum-based detection.	Expression increases with the advancing stage; in sputum, shows higher sensitivity vs. cytology for lung cancer diagnosis.	[113,122]
miRNA-942	NSCLC early diagnosis.	Significantly upregulated, outperforms traditional markers (CEA, CYFRA21-1, SCCA).	[114]
miRNA-601	NSCLC early diagnosis.	Significantly upregulated, outperforms traditional markers, and combining with miR-942 improves early detection.	[114]
miRNA-21-5p	Male lung squamous cell carcinoma (LUCS); also part of a protein–miRNA panel.	Differential expression between early- and late-stage LUCS. In a separate study, combined with a 4MP panel to enhance sensitivity at 95% specificity.	[108,115]
miRNA-181-5p and 155-5p	Male LUCS.	Differential expression between early- and late-stage disease.	[115]
miRNA-3692-3p	NSCLC early diagnosis.	Upregulated in NSCLC but not correlated with treatment response or survival.	[116]
miRNAs-199a-3p, chr17_10932, 148a-3p, 210-3p, chr1_1402, 378d, and 138-5p	LUAD especially with ground-glass nodules (GGNs).	Overexpressed and part of a 7-miRNA early-detection panel.	[117,119]
miRNA-146a-5p	Early detection of lung cancer.	The addition to the mRNA panel improves AUC from 0.66 to 0.71 (diagnostic accuracy).	[118]
miRNA-301a-5p	Early-stage LUAD and LUCS.	Potential biomarker; upregulated in early disease.	[119]
miRNA-205	Early-stage squamous cell lung cancer.	Overexpressed in sputum of squamous cell lung cancer patients; offers good early detection accuracy.	[123]
miRNA-324-3p	Early detection of stage I LUCS.	Significantly upregulated; AUC = 0.79 alone and improves to 0.89 when combined with miR-1285; independent prognostic indicator.	[124]
miRNA-1285	Early detection of stage I LUCS.	Significantly downregulated; combining with miR-324-3p improves diagnostic accuracy.	[124]
miRNAs-320a-3p, 92a-3p, and 140-3p	Early detection of lung cancer.	Combined with a 4-protein marker panel (4MP) to improve sensitivity by 19% at 95% specificity.	[108]

### 3.3. Prognostic Biomarkers—miRNA Signature Correlated with Patient Outcomes

Some types of miRNA have been associated with certain clinical outcomes (Table 6). miRNAs-21, 31, and let-7 expression were increased in lung cancer patients with lymph node metastasis than those without lymph node metastasis. Moreover, the patient’s median survival time is correlated with miRNA expression. It is worth mentioning that median survival time is longer when miRNAs-21, 31, and let-7 expression is low [82]. Lower expression of miRNA-148a is related to lymph node metastasis and advanced clinical stage. Moreover, patients with low expression of miRNA-21 demonstrate shorter disease-free survival (DFS) and overall survival (OS) according to the Kaplan–Meier analysis [125]. Moreover, reduced miRNA-21 expression suppressed the growth and cell cycle progression of A549 cells [126]. Furthermore, increased miRNA-let7 expression suppressed the proliferation of the A549 lung cancer cell line [127]. However, these in vitro findings require further investigation through future studies before they can be translated into therapeutic applications.

miRNA-182-5p overexpression is strongly associated with lymph node metastasis in NSCLC and LUAD patients. However, the Kaplan–Meier curve does not demonstrate significant survival outcome differences among the aforementioned patients [77]. Lower miRNA-30c concentration during or before treatment is related to shorter OS and RFS with the AUC at 0.872 [128]. miRNA-30a-5p expression is decreased in LUAD. Furthermore, the TN stage, pathologic stage, residual tumor, primary therapy outcome, and OS of LUAD patients are related to the miRNA-30a-5p expression [129]. It is worth mentioning that miRNA-30a-5p shows a negative correlation with *BCL-2* and is associated with favorable clinical outcomes in NSCLC patients [130]. Lung cancer patients after lung resection are less likely to develop metastases when has-miRNA-197 is upregulated. Furthermore, the has-miRNA-197 expression is close to control in patients who develop metastasis [131].

miRNAs-19a-3p, 126-5p, 556-3p, 671-5p, 937-3p, 4664-3p and 4746-5p expression is associated with OS in LUAD patients. Moreover, among high-risk groups, mortality was higher [132]. Decreased miRNA-125b-5p expression is correlated with poor prognosis among LUAD patients [133]. miRNAs-320a,25-3p, and 148a-3p expression is significantly related to the stage of tumor in NSCLC patients [134]. Moreover, miRNA-25-3p is related to shorter OS in lung cancer patients [135]. Vucic et al. have shown that 31 miRNAs except miRNA-135b are associated with LUAD patients’ survival regardless of tumor stage. Among these 31 microRNAs strongly linked to patient outcomes were miRNAs-let-7g, 21, 1, and 138 [136]. 

miRNAs-9-2, 125, and 193a methylation is related to NSCLC patients OS. Patients with the highest level of methylated miRNA-127 have 17 months shorter survival time compared to 51.6 months survival time for the low-risk group [137]. miRNAs-148b, 365, 32, 375, 21, 125b, and 155 display prognostic ability in NSCLC patients. Furthermore, patients with high-risk miRNA signatures have shorter median OS. It is worth noting that miRNA signature composed with tumor stage information exhibited enhanced prognostic accuracy [138]. Moreover, decreased miRNA-375 expression is associated with worse survival in NSCLC patients [139]. 

miRNAs-let-7f, 30e-3p, and 20b are related to the NSCLC stage and lymph node metastases. Moreover, elevated plasma levels of miRNAs-let-7f and 30e-3p were associated with unresectable tumors, indicating advanced-stage disease. miRNA-let-7f is related to lower OS in patients with nonresectable and more malignant NSCLC, which suggests that miRNA-let-7f might be linked with poor prognosis. Moreover, the elevated plasma level of miRNA-30e-3p is correlated with shorter DFS [140]. NSCLC patients with higher miR-3195 expression had significantly longer OS. Moreover, multivariate analysis identified miR-3195 as an independent prognostic factor for OS [141]. 

miRNA-424 expression showed no difference between NSCLC tumors and healthy lung tissues but was significantly upregulated in tumors from patients at advanced clinical stages. High miRNA-424 expression was strongly associated with advanced stages, aggressive metastasis, and shorter survival and was identified as a potential independent prognostic marker in NSCLC according to Cox regression analysis [81]. Tumors that recurred had significantly lower miRNA-221 expression compared to those that did not recur. Furthermore, higher miRNA-221 expression in tumor tissue relative to adjacent normal lungs correlated with non-recurrence. This finding suggests that miRNA-221 expression in resected NSCLC may serve as a marker to identify patients at high risk of post-surgical relapse [142]. NSCLC patients with high serum miRNA-942 and miRNA-601 levels had the poorest outcomes, while those with low levels had the best prognosis. Multivariate analysis confirmed these miRNAs as independent prognostic factors for NSCLC [114]. 

It is worth noting that miRNAs-6777-5p, 6780a-5p, and 877-5p signatures from exhaled breath condensate can serve as prediction biomarkers in lung cancer patients at 0.8 individual AUCs [83]. Joerger et al. have shown that miRNA-665 is the strongest predictive marker for tumor shrinkage in patients with advanced non-squamous NSCLC receiving bevacizumab/erlotinib followed by platinum-based chemotherapy. miRNA-223 is significantly linked to time to disease progression (TTP) in patients following bevacizumab/erlotinib treatment. Patients receiving bevacizumab/erlotinib treatment and chemotherapy with increased miRNA-223 expression exhibit worse outcomes. Moreover, higher miRNA-223 expression was observed in patients with disease stabilization [143]. 

High expressions of miRNA-128 and 155 were correlated with shorter OS in NSCLC patients. Furthermore, increased miRNAs-21, 128, 155, and 181a are associated with worse outcomes in squamous cell NSCLC patients [144]. Elevated plasma miRNA-202 expression was associated with disease progression and independently predicted shorter PFS and OS in NSCLC patients treated with first-line platinum-based chemotherapy. In subgroup analyses, high miRNA-202 predicted shorter OS in the non-squamous subgroup, while high miRNA-26a was associated with worse OS in the squamous subgroup [145] miRNA-150 and 886-3p signature predicted OS and PFS in early-stage SCLC treated with surgery and adjuvant chemotherapy, with high-risk signatures correlating with poorer outcomes. These miRNAs, downregulated in SCLC tissues, serve as independent prognostic biomarkers for patient stratification and treatment optimization [146]. 

miRNAs-143, 100, 101-1, 101-2, 182, 183, 205, 21, 30a, and 30d expression identified through machine learning significantly correlated with survival in LUSC patients and are strong predictors of outcomes [147]. Elevated miRNAs-155-5p and 223-3p and lower miRNA-126-3p expression are related to poor DFS in LUAD patients. Moreover, higher miRNAs-20a-5p and lower miRNAs-152-3p and 199a-5p expression are linked to lower DFS in LUCS patients [148]. Reduced expression of miRNA-181a-5p and 630 in NSCLC tissue and plasma was associated with improved prognosis, including longer PFS and OS. Thus, circulating miRNAs-181a-5p in plasma and tumor tissue levels may serve as significant independent prognostic factors for NSCLC outcomes [149].

**Table 6:** The summary of miRNAs correlated with patients’ outcomes.

miRNA/miRNAs	Connection with Patient Outcomes	Characteristic Features	Ref.
miRNA-21	Increased expression in lung cancer patients with LN metastasis. Low expression correlates with a longer median survival time. However, higher expression correlates with shorter DFS and OS.	Contradictorily impact on patient outcomes according to different references.	[82,124,143]
miRNA-31	Increased expression in lung cancer patients with LN metastasis; lower expression correlates with longer median survival.	Associated with metastasis and shorter survival when overexpressed.	[124]
miRNA-let-7	Increased expression in lung cancer patients with LN metastasis; lower expression correlates with longer median survival.	Higher miRNA-let-7 is linked to metastasis.	[124,135]
miRNA-25-3p	Linked to shorter OS in lung cancer patients.	May be grouped in panels for prognostic or diagnostic use.	[134]
miRNA-182-5p	Overexpression is strongly associated with LN metastasis in NSCLC/LUAD, but no significant survival difference on Kaplan–Meier curves.	miRNA-182-5p may be more useful as a metastatic marker than a clear prognostic factor for OS or DFS.	[125]
miRNAs-320a, 25-3p, and 148a-3p	Expression levels are significantly related to tumor stage in NSCLC.	miRNA-25-3p especially is related to shorter OS in lung cancer.	[133,134]
miRNAs-let-7f, 30e-3p, and 20b	Related to NSCLC stage and LN metastases. Elevated expression of miRNAs-let-7f and 30e-3p are correlated with unresectable tumors. Elevated miRNA-let-7f is linked to lower OS in advanced NSCLC; high miRNA-30e-3p expression is associated with shorter DFS.	miRNA-let-7f is considered a poor prognostic indicator for nonresectable NSCLC.	[139]
miRNA-424	Elevated expression is associated with tumor stage as well as shorter survival.	Associated with aggressive metastasis and advanced clinical stage.	[141]
miRNA-942 and 601	High serum levels are associated with worse outcomes in NSCLC.	Elevated expression is linked to a worse prognosis.	[114]
miRNA-223	Significantly linked to time to disease progression (TTP); elevated miRNA-223 expression is associated with worse outcomes.	May predict treatment response and TTP.	[83]
miRNA-128 and 155	High expression is associated with shorter OS in NSCLC.	Elevated miRNAs-21, 128, 155, and 181a are linked to worse outcomes in NSCLC patients.	[143]
miRNA-181a	Increased expression is associated with worse outcomes in squamous cell NSCLC.	-	[143]
miRNA-202	Elevated expression is correlated with disease progression; predicts shorter PFS and OS in NSCLC; high miR-202 is associated with shorter OS in non-squamous patients.	Independent predictor of poor outcome.	[144]
miRNA-26a	High expression is correlated with worse OS in squamous NSCLC patients.	Histological-specific association with poor prognosis.	[144]
miRNA-181a-5p, and 630	Reduced expression in NSCLC tissue is linked to longer PFS and OS.	Elevated levels are associated with worse outcomes; serve as independent prognostic markers in NSCLC.	[148]
miRNA-20a-5p	Higher expression is linked with lower DFS in LUCS patients.	Potentially prognostic biomarker in LUCS.	[147]
has-miRNA-197	Upregulation correlates with a lower risk of metastases post-lung resection.	Expression in metastasis-developing patients remains near control levels, suggesting a protective role.	[130]
miRNA-221	Lower expression in recurrent tumors; higher expression correlates with no recurrence.	May help identify patients at high risk of post-surgical relapse.	[81]
miRNA-3195	Higher expression is associated with longer OS in NSCLC; identified as an independent prognostic factor.	May have a prognostic role.	[140]
miRNA-665	Strongest predictive marker for tumor shrinkage in advanced non-squamous NSCLC patients treated with bevacizumab/erlotinib chemotherapy.	High expression might be linked with better tumor response.	[83]
miRNA-148a	Lower expression is related to LN metastasis and advanced clinical stage.	Low expression correlates with more aggressive disease.	[82]
miRNA-30c	Lower concentration is associated with shorter OS and RFS.	Potentially useful as a predictive biomarker for treatment response and survival.	[77]
miRNA-30a-5p	Decreased in LUAD; associated with TN stage, pathologic stage, residual tumor, primary therapy outcome, and OS.	Negatively correlates with *BCL-2*. Associated with favorable outcomes.	[128,129]
miRNA-125b-5p	Decreased expression is correlated with poor prognosis in LUAD.	Potentially tumor-suppressive role in LUAD.	[132]
miRNA-375	Decreased expression is correlated with worse survival in NSCLC.	Often discussed as a tumor-suppressor miRNA in lung cancer.	[138]
miRNA-126-3p	Lower expression is associated with poor DFS in LUAD patients.	Often considered a tumor suppressor in various lung cancer contexts.	[147]
miRNA-152-3p and 199a-5p	Lower expression is associated with lower DFS in LUCS.	Decreased expression is associated with worse outcomes in squamous cell subtype patients.	[147]
miRNAs-9-2, 125, 193a	Methylation status correlates with OS in NSCLC.	Higher methylation is often associated with worse outcomes.	[136]
miRNA-127	Patients with the highest level of methylated miR-127 have a longer median survival.	Increased methylation is associated with shorter survival.	[137]
miRNAs-19a-3p, 126-5p, 556-3p, 671-5p, 937-3p, 4664-3p, and 4746-5p	Expression levels are associated with OS in LUAD; high-risk groups show higher mortality.	Part of a multi-miRNA prognostic signature specific to LUAD.	[131]
31 miRNAs (except miRNA-135b)	All are associated with LUAD patient survival regardless of tumor stage	Part of a broad prognostic signature in LUAD.	[135]
miRNAs-148b, 365, 32, 375, 21, 125b, and 155	Display prognostic ability in NSCLC; high-risk signature is linked to shorter median OS. Decreased miR-375 is correlated with worse survival.	Combining miRNA signatures with tumor stage enhances prognostic accuracy.	[138]
miRNA-150 and 886-3p	miRNAs signature may predict OS and PFS in early-stage SCLC patients treated with surgery and adjuvant chemotherapy.	Downregulated in SCLC tissues. Potentially serve as independent prognostic biomarkers for patient stratification.	[145]
miRNAs-143, 100, 101-1, 101-2, 182, 183, 205, 21, 30a, and 30d	Identified via machine learning as significantly correlated with survival in LUSC; strong predictors of patient outcome	miRNA signature identified via machine learning is associated with LUSC prognosis.	[146]
miRNA-155-5p and 223-3p	Elevated expression is linked to poor DFS in LUAD patients.	DFS may be lower when this miRNA signature is overexpressed.	[147]
miRNA-6777-5p, 6780a-5p, and 877-5p	Expression in exhaled breath condensate may serve as potential prediction biomarkers in lung cancer.	Might be used as a noninvasive diagnostic in the future.	[142]

### 3.4. Predictive Biomarkers—The Role of miRNAs in Predicting Treatment Response

Shi et al. demonstrated that serum levels of miRNAs-25, 145, and 210 might be useful in predicting treatment with pemetrexed response in patients with advanced NSCLC. Nonetheless, this study is limited by a small sample size. Thus, further investigations are required [150]. The backbone of inoperable stage III NSCLC treatment is concurrent chemoradiotherapy (cCRT). However, resistance mechanisms such as autophagy up-regulation may make this therapy ineffective. miRNAs-375, 200c, and 30cin extracellular vesicles (EV) expression were lower among non-responders to CRT. The aforementioned miRNAs influence the phosphatidylinositol-mediated signaling pathway (PI3K) involved in autophagy regulation thus and associated with immune regulation. These findings suggest that miRNAs may be useful in predicting treatment response to PI3K inhibitors or cCRT [128]. 

It is worth mentioning that miRNA levels may correlate to treatment response (Table 7). NSCLC patients who responded to chemotherapy exhibited higher miR-1249-3p expression compared to non-responders [141]. The seven miRNAs-101-2, 139, 182, 183, 190, 326, and 944 signatures are correlated with LUCS patient outcomes. According to the Kaplan-Meier test, high-score LUSC patients have lower OS [151]. miRNAs-142-3p and 29b expression are increased when early-stage LUAD recurrence occurs. However, miRNA-142-3p is more predictable than miRNA-29b regarding clinical parameters. Moreover, miRNA-142-3p expression is upregulated when adjuvant therapy has a poor outcome [152]. 

miRNA-200b expression in NSCLC patient specimens is negatively correlated with programmed death-ligand 1 (PD-L1), which might have clinical utility. Moreover, PD-L1 expression can be used to predict response to immune checkpoint inhibitors (ICIs) using tumor proportion score (TPS). However, a high PD-L1 TPS (≥50%) has demonstrated only a 44.8% response rate to pembrolizumab monotherapy, suggesting that PD-L1 TPS alone is an inadequate biomarker [153]. miRNA-30a-5p increases NSCLC sensitivity to paclitaxel in vitro and in vivo. Moreover, miRNA-30a-5p enhances the sensitivity of NSCLC cells to paclitaxel by promoting apoptosis through the suppression of *BCL-2* [130]. 

miRNA expression patterns in NSCLC cells cultured in 3D suggest that circulating miRNA-10a-3p could serve as a novel non-invasive biomarker for predicting the short-term prognosis of NSCLC patients [154]. miRNAs alterations were found among NSCLC patients harboring Del19 and L858R EGFR mutations. In patients carrying the Del19 mutation, the expression of 76 miRNAs was increased, while the expression of three miRNAs was decreased [155]. An overall survival benefit has been observed in patients with Del19-mutated tumors but not in those with L858R mutations following treatment with afatinib, an irreversible ErbB family inhibitor [156]. Thus, different miRNA expressions might be biomarkers that impact clinical outcomes. miRNAs-92a-2, 147, and 574-5p are importantly linked with chemoresistance. Elevated tumor levels of miR-92a-2 are linked to chemoresistance and reduced survival in SCLC patients. miRNA-92a-2 could serve as a potential biomarker for identifying SCLC patients at risk of developing de novo chemoresistance. However, additional validation in independent sample cohorts is needed [157]. 

miRNAs-105-5p and 767-5p are the most significant for survival prediction in LUAD patients treated with ICI. Patients with high levels of these miRNAs showed poorer survival compared to those with lower expression levels, distinguishing long from short therapy responders [119]. Levels of miRNAs-let-7f and 30e differentiated patient groups by disease stage and surgical eligibility. Therefore, miRNAs-let-7f and 30e levels distinguished between patients with resectable lung tumors and those with non-resectable lung tumors [140]. miRNA-455-5p and PD-L1 exhibit inverse correlation in NSCL patients. miR-455-5p regulates cisplatin resistance as a result of PD-L1 expression. Thus, it might be a predictive biomarker of chemotherapy outcome [158]. Elevated miRNA-486-5p serum level is associated with a longer time to progression of NSCLC after platinum-based chemotherapy. This finding was assessed using Cox hazard regression analysis [159]. High miRNA-34a expression was linked to shorter OS in NSCLC patients treated with platinum-based chemotherapy and gemcitabine, while high miRNA-224 expression correlated with shorter OS in patients receiving chemotherapy combined with radiotherapy. Thus, those miRNAs might be predictive biomarkers in NSCLC patients [160]. Ectopic miRNA-34a reduces proliferation and invasion of KrasG12D/+;Trp53R172H/+ lung epithelial cells in vitro, which makes it potentially useful in lung cancer treatment [161]. Fan et al. have identified 27 serum miRNAs differentially expressed between responders and nonresponders to immunotherapy, with miRNAs-93, 138-5p, 200, 27a, 424, 34a, 28, 106b, 193a-3p and 181a significantly increased in responders. NSCLC patients with high expression of these miRNAs showed improved progression-free survival (PFS) 6.25 vs. 3.21 months and OS, highlighting their potential as predictors of response and survival after checkpoint inhibitor therapy [162]. miRNAs-22, 24, and 34a expression are increased in NCLC patients, and their expression is correlated to pemetrexed response. Moreover, miRNA-22 upregulation is associated with developing progressive disease [163].

**Table 7:** The summary of the predictive role of miRNAs in lung cancer patients.

miRNA/miRNAs	Predictive Role	Characteristic Features	Ref.
miRNA-25, 145 and 210	Predicts response to pemetrexed in advanced NSCLC.	Serum levels, together with miRNA-145 and miRNA-210, may help identify which patients respond to pemetrexed. However, further validation is needed due to a small sample size.	[150]
miRNA-1249-3p	Predicts chemotherapy response in NSCLC.	Higher expression in responders compared to non-responders, indicating potential use as a biomarker to differentiate treatment response in NSCLC.	[141]
miRNA-30a-5p	Predicts sensitivity to paclitaxel in NSCLC.	Increases NSCLC sensitivity to paclitaxel in vitro and in vivo by promoting apoptosis through *BCL-2* suppression, suggesting it could be a useful biomarker for chemotherapy.	[130]
miRNA-10a-3p	Novel non-invasive biomarker for short-term NSCLC prognosis.	Identified in NSCLC cells cultured in 3D. miRNA-10a-3p circulating levels may help predict short-term survival outcomes.	[154]
miRNA-200b	Potential immunotherapy predictor due to correlation with PD-L1.	Negatively correlated with PD-L1 expression in NSCLC tissues thus may improve predictive value beyond PD-L1 tumor proportion scores (TPS) for selecting patients for immune checkpoint inhibitor therapy.	[153]
miRNA-let-7f and 30e	Distinguish resectable vs. non-resectable NSCLC.	Elevated plasma levels correlate with advanced disease and non-surgical cases, helping to determine surgical eligibility.	[140]
miRNA-455-5p	Predicts cisplatin resistance.	Inversely correlated with PD-L1 expression and regulates cisplatin resistance in NSCLC, suggesting it could be a predictive biomarker of chemotherapy outcomes.	[158]
miRNA-486-5p	Predicts progression after platinum-based chemotherapy in NSCLC.	Elevated serum levels are associated with a longer time to progression, as confirmed by Cox hazard regression analysis, suggesting a potentially more favorable response.	[159]
miRNA-34a	Predicts survival in NSCLC patients on platinum and gemcitabine chemotherapy.	High miRNA-34a expression is linked to shorter OS in patients treated with chemotherapy and radiotherapy. Nonetheless, immunotherapy responders exhibit elevated miRNA-34a expression, which correlated with improved outcomes.	[160,162]
miRNA-224	Predicts OS in NSCLC patients receiving chemotherapy with radiotherapy.	High miRNA-224 expression correlates with shorter OS in combined chemo-radiotherapy.	[160]
miRNAs-93, 138-5p, 200, 27a, 424, 28, 106b, 193a-3p, and 181a	Predict immunotherapy response and survival; higher miRNA expression in responders is associated with better PFS and OS.	Identified in a 27-miRNA panel that distinguishes responders from nonresponders to checkpoint inhibitors. Patients with high expression experience longer PFS and improved OS.	[162]
miRNA-22, 24, and 34a	Correlate with pemetrexed response in NSCLC; miRNA-22 upregulation linked to progressive disease.	Upregulated in patients receiving pemetrexed. miRNA-22 is specifically associated with progressive disease, while higher miRNA-24 and miRNA-34a levels correlate with pemetrexed sensitivity. However, miRNA-34a is correlated with poor outcomes in chemotherapy patients.	[163]
miRNA-142-3p	Predicts early-stage LUAD recurrence and poor outcome with adjuvant therapy.	More predictive than miRNA-29b for LUAD recurrence and upregulated in patients whose adjuvant therapy outcome is poor, indicating its potential as a biomarker for relapse risk.	[152]
miRNA-29b	Associated with early-stage LUAD recurrence.	Elevated levels during LUAD recurrence, though less predictive than miRNA-142-3p, may help identify patients at higher risk of early relapse.	[152]
miRNA-105-5p and 767-5p	Predict immunotherapy survival outcomes in LUAD.	High levels are associated with poorer survival in LUAD patients treated with immune checkpoint inhibitors. It might be used to distinguish long responders from short responders.	[119]
miRNA-101-2, 139, 182, 183, 190, 326, and 944	Predict outcomes in LUSC.	A seven-miRNA signature correlated with lower overall survival in high-score LUSC patients, suggesting its possible prognostic utility in this subtype.	[151]
miRNA-92a-2	Predicts chemoresistance in SCLC.	Elevated levels are linked to chemoresistance and reduced survival, suggesting its use as a possible biomarker for de novo resistance in SCLC.	[157]
miRNA-147 and 574-5p	Linked to chemoresistance in lung cancer.	Elevated miRNA-147 and 574-5p tumor levels are associated with treatment resistance and reduced survival.	[157]
miRNA-375, 200c and 30c	Predicts response to concurrent chemoradiotherapy (cCRT) or PI3K-targeted therapy.	Lower expression in non-responders to cCRT, influences phosphatidylinositol-mediated signaling and autophagy and may also be relevant for immune regulation and treatment resistance.	[128]

## 4. miRNAs as Therapeutic Targets

miRNAs represent a promising yet complex and still extensively researched therapeutic option for the treatment of various cancers, including different types of lung cancer. Current studies are based on the observation that cancer tissues exhibit a significant reduction in tumor-suppressive miRNAs and an increase in tumor-promotive (oncogenic) miRNAs [164].

### 4.1. miRNA Replacement Therapy

The group of ‘tumor suppressor genes’ includes those whose miRNA products inhibit the expression of oncogenes that promote tumor development, growth, and invasiveness. Reduced expression of these genes is a therapeutic target for replacement therapies, which aim primarily at their restoration, regulation of disrupted cellular functions, and, finally, inhibiting tumor onset and progression [165,166]. The exploration of tumor-suppressive miRNAs has led to the identification of several promising candidates that may serve as a foundation for developing new therapeutic strategies. Among these are miR-30a, miR-30b, miR-30d, miR-99a, miR-124, miR-144, miR-195, miR-223, miR-335, and miR-363 I miR-451 [167]. Recent research has particularly emphasized the therapeutic potential of three key groups: miR-34a, the let-7 family, and the miR-29 family.

miR-34a is a well-established tumor suppressor miRNA controlled by the TGF- β signaling path in lung cancer [168]. It is known for its ability to modulate pathways that maintain cellular balance, inhibit abnormal cell growth, and preserve genomic integrity. It also plays a critical role in suppressing epithelial-to-mesenchymal transition (EMT), thereby reducing cancer cell spread and invasion [14,169,170]. Notably, miR-34a was the first miRNA to enter clinical trials as a therapeutic agent [164,169]. 

The let-7 family of miRNAs is another example of strongly-characterized tumor suppressors with significant roles in regulating cancer progression by targeting multiple oncogenes and inhibiting crucial mitotic pathways [14,165]. For instance, let-7a suppresses the growth of lung cancer cells by targeting oncogenes like K-Ras and c-Myc. Furthermore, let-7b directly targets the BRF2-mediated MAPK/ERK pathway, inhibiting the proliferation and metastasis of lung adenocarcinoma (LUAD) cells. Let-7c contributes by reducing the migration and invasion of cancer cells [164,165].

The miR-29 family, comprising miR-29a, miR-29b, and miR-29c, has been extensively studied in various cancers, including lung squamous cell carcinoma (LUSC). Expression profiles consistently show significant downregulation of miR-29s in cancerous tissues compared to healthy ones [171]. Loss of miR-29 expression has been linked to increased cancer cell invasion and migration, primarily through the upregulation of oncogenic targets like lysyl oxidase-like 2 (LOXL2). Overexpression of LOXL2 has been correlated with poor prognosis in cancers such as gastric, breast, and squamous cell carcinomas. Restoration of miR-29s in cancer cell lines has demonstrated significant inhibition of migration and invasion [171,172].

### 4.2. Strategies to Restore Tumor-Suppressive miRNAs in Lung Cancer

Restoring tumour-suppressive miRNAs in lung cancer involves several innovative strategies, such as miRNA mimics, miRNA precursors, as well as miRNA-expressing plasmids. miRNA mimics (double-stranded molecules) are designed to simulate endogenous miRNA and bind to the target gene mRNA. As a result of chemical modifications to miRNA mimics, miRNA agomirs have been developed, demonstrating enhanced activity and stability. Pre-miRNAs (single-stranded RNA) molecules were designed to resemble mature miRNAs. They might be delivered into cells via transfection or electroporation, where they are processed into functional miRNAs. Another promising approach uses miRNA-expressing plasmids, which not only upregulate specific miRNAs but also include fluorescent markers, enabling researchers to monitor their expression and distribution [173].

### 4.3. Approaches to Inhibit Oncogenic miRNAs

Oncogenic miRNAs, also known as oncomiRs, play a critical role in cancer progression by repressing tumor-suppressor genes and promoting cell proliferation, metastasis, and drug resistance. Targeting these miRNAs has emerged as a promising therapeutic approach in cancer treatment, aiming to restore normal cellular function and gene regulation. Strategies to inhibit oncomiRs are based on exogenous miRNA mimics supply and they include artificial inhibitors such as antagomiRs and miRNA sponges [14,165,170]. Throughout different studies, there have been some potential oncogenic miRNAs identified, with miR-21 and miR-31 being the most researched ones [52,164,167]. miR-21 is a well-known oncomiRNA that may contribute to tumor progression, invasiveness, and metastasis by suppressing PTEN, TGF-β, and PDCD4 [170]. Its overexpression is observed in a variety of tumors, for example, breast cancer, ovarian cancer, colon cancer, and lung cancer [14]. miR-31 functions as an oncomiR by inhibiting the expression of specific tumor suppressor genes, such as *LATS2* and *PPP2R2A*, in lung cancer. Knockdown of miR-31 significantly reduced cell growth and proliferation in both murine and human lung cancer cell lines. Additionally, engineered repression of miR-31 resulted in reduced clonal growth and tumorigenicity in vivo [52].

#### 4.3.1. AntagomiRs

AntagomiRs are chemically engineered oligonucleotides (DNA or DNA analogs) designed to inhibit the function of specific microRNAs by directly binding small RNA molecules in the RNA-induced silencing complex (RISC) [165,174]. This mechanism effectively disrupts the post-transcriptional regulation mediated by miRNAs, offering a powerful approach to silencing oncomiRs that promote tumorigenesis. Although this method may seem promising in lung cancer therapy, it only allows the inhibition of one specific and well-known oncogenic miRNA. Other drawbacks of this method include low bioavailability and poor cellular uptake. Additionally, enzymes acting both intracellularly and extracellularly cause the degradation of oligonucleotides over time, meaning that effective therapy would require repeated administrations [175].

#### 4.3.2. miRNA Sponges

miRNA sponges present a novel approach in strategies to inhibit oncomiRs despite occurring physiologically in various plants, animals, and humans. Their main purpose is to capture and bind sequence-specific miRNAs or the entire miRNA families sharing the same seed regions [175]. These sponges are typically composed of a non-toxic gene with multiple miRNA-binding sites inserted into its 3′ untranslated region (3′ UTR) [174]. The binding sites are usually arranged in tandem repeats to increase the likelihood of miRNA recognition and binding. Once introduced into cells, typically through viral or non-viral vectors, miRNA sponges bind to endogenous miRNAs, preventing their interaction with natural mRNA targets [175]. The advantage of miRNA sponges over anti-miRNA oligonucleotides lies in their ability to simultaneously target multiple miRNAs, which are often involved in complex regulatory networks.

### 4.4. Challenges and Opportunities

#### 4.4.1. Delivery Systems for miRNA-Based Therapies

Effective methods of delivering miRNA-based agents are crucial in new types of anticancer therapies. Modern systems (Table 8) are designed to protect therapeutic molecules from degradation and precisely target the cancer cells. Unfortunately, these are the limitations that miRNA-based therapies are still facing. Two primary categories of delivery methods are viral and non-viral systems—each having benefits and drawbacks that need well-thought design and application [176,177].

##### Viral-Based Vectors

Viral vectors, including adenoviruses, retroviruses, lentiviruses, and adeno-associated viruses (AAV), are highly efficient at gene transfection and provide sustained expression in target tissues. The mentioned viruses are widely used in experimental models, making them the most common vectors in animal research [175]. However, their clinical application presents numerous challenges and difficulties depending on the vector [170,173,175,176].

AAVs have gained significant attention due to their extremely high efficiency, making them a promising tool in miRNA therapies. However, their clinical application presents major challenges. Adenoviral vectors can trigger a strong immune response, which is associated with prior exposure to wild-type strains of the virus. Adverse effects include inflammation, fever, organ failure, and, in extreme cases, death. It is important to note that even if the patient does not experience severe systemic complications, the immune response can significantly impair the effectiveness of the therapy and prevent repeated administration [173,175]. On the other hand, AAVs emerge as a better alternative to adenoviruses due to their lower immunogenic potential. Recent studies focus on improving tissue specificity, which would ultimately enhance drug efficacy and reduce the risk of off-target effects [178].

The primary advantage of retroviral and lentiviral vectors is their ability to introduce genetic material that integrates into the host genome, enabling stable gene expression, which is crucial for long-term therapy. However, this capability is not without risk—there is a legitimate concern that integration into transcriptionally active regions may lead to insertional mutagenesis and, consequently, carcinogenesis [175,176,178].

##### Non-Viral-Based Vectors

Non-viral delivery systems, on the other hand, present safer alternatives by reducing immune responses and avoiding nuclease degradation. However, these options usually lack good gene transfer efficiency, especially for in vivo applications [164,175,176]. These systems use a variety of carriers, including liposomes, polymers, nanoparticles, and extracellular vesicles, to deliver therapeutic molecules such as miRNA mimics or inhibitors.

Lipid nanoparticles, like liposomes, are a carefully designed combination of lipids that protect RNA from enzymatic degradation and facilitate its cellular uptake, making them widely used in transporting miRNA in vivo. The broad variety and high availability of cationic liposomes make them appealing delivery systems for research applications [173,176,178]. They are relatively easy for host cells to capture; however, they exhibit high tropism for liver cells and low tissue specificity, which may lead to interactions with unintended cells and off-target effects. Another challenge is the low efficiency of genetic material delivery and susceptibility to phagocytosis. For this reason, recent research on lipid nanoparticles focuses on incorporating various functional groups and ligands to enhance the effectiveness, stability, and specificity of therapeutic molecules, enabling targeted binding to specific cells, such as cancer cells [176,178]. For example, a liposomal carrier was used in a clinical trial investigating the use of miRNA-34 in patients with advanced solid tumors. Although the trial was terminated prematurely due to the deaths of four patients, researchers did not attribute the drug’s toxicity to the delivery vector [179].

Polymeric vectors are extensively studied carriers in miRNA-based therapies. They offer key benefits such as biodegradability, low immunogenicity, as well as the flexibility to be synthesized and modified according to research requirements [177,178]. The most commonly used are polyethylenimines (PEIs), which are positively charged molecules rich in amine groups. They strongly bind to RNA, providing protection against degradation and enhancing cellular uptake. Unfortunately, they exhibit cytotoxicity, which necessitates further refinement of this delivery method [173,176]. The solution to this problem could be combining PEIs with other polymers, such as PEG (polyethylene glycol), PLGA (poly(lactic-co-glycolic acid)), or PLL (poly-L-lysine), which increases the biocompatibility and stability of the particles, as well as improves miRNA delivery efficacy and overall performance [176,177].

##### Extracellular Vesicles

The heterogeneous group of extracellular vesicles includes exosomes, microvesicles, and apoptotic bodies. The advantages of this group of vectors are their low immunogenicity and cytotoxicity, as well as high efficiency in delivery [173,177]. Exosomes are physiologically secreted by most cells in the body, found in almost all body fluids, and serve as natural carriers of RNA, making them attractive vectors for miRNA-based therapies [177]. However, a significant limitation is the high cost of their mass production—exosomes are obtained by engineering cells that produce excess amounts of specific miRNA and packaging them into exosomes or by enriching already collected exosomes with additional copies of of miRNA [173,176]. Microvesicles and apoptotic bodies also carry miRNA and, in theory, could be used to develop modern therapeutic methods. However, this would require significant time and financial investments to thoroughly understand these molecules [176,177].

### 4.5. Current Clinical Trials and Prospects

The exploration of miRNA-based therapies for lung cancer has shown promising results but also presents significant challenges (Table 9). Clinical trials aimed at leveraging miRNAs to treat non-small cell lung cancer, including LUSC and LUAD, have highlighted the potential of these molecules in modulating tumor growth, metastasis, and immune response. Several miRNAs, such as miR-34, miR-155, miR-21, and miR-16, have been investigated for their therapeutic roles in clinical trials [165,170].

MiR-34, known for its tumor-suppressive properties, has been the focus of multiple clinical trials. A Phase I trial involving MRX34, a synthetic miR-34a mimic delivered via liposomal nanoparticles, was initially promising but was halted due to severe immune-related adverse events. These events, including sepsis, hypoxia, cytokine release syndrome, hepatic failure, and patients’ deaths, underscored the significant challenge of effectively and safely delivering miRNAs to patients without causing unintended immune responses [179]. Despite these setbacks, MRX34 has been explored in subsequent trials, showing great potential. Other studies have combined miR-34a with chemotherapy, demonstrating an ability to inhibit tumor growth and improve survival outcomes, although issues with delivery systems and immune toxicity remain persistent challenges [165,168,170].

Other miRNAs, like miR-16 and miR-155, have also been explored as therapeutic targets. For instance, MesomiR-1, a liposomal miR-16 mimic, has been studied in thoracic malignancies, including NSCLC in advanced stages. This therapy showed promising results in early-phase trials, achieving acceptable safety in patients [169]. miRNA-155 has been extensively studied in clinical trials due to its dual role in immune regulation and cancer progression. Downregulation of miR-155 has been linked to increased tumor growth, heightened aggressiveness, and altered responses to therapies such as tamoxifen and cisplatin. However, targeting miR-155 in therapy has proven challenging, as its complex biological interactions often lead to unintended consequences, complicating its clinical application [170]. Similarly, miR-21 entered the third phase of clinical trials, although its results did not meet the anticipated endpoints, proving that engineering a new molecular method of lung cancer treatment is a complex challenge [170].

### 4.6. Hurdles in miRNA-Based Therapies in Lung Cancer

Despite significant progress in understanding the mechanisms of miRNA action in cells, including their involvement in carcinogenesis, their application in lung cancer therapy still faces numerous challenges. One of the greatest difficulties seems to be the precise characterization of the multifaceted effects of individual molecules and the systematization of knowledge regarding their influence on specific genes and pathways. Undoubtedly, this process will be both costly and time-consuming, requiring various studies conducted in vitro, in vivo on animal models, and, ultimately, in humans. As demonstrated by research on MRX34, even despite promising results in animal models, clinical trials are associated with significant responsibility and a high risk of errors [179].

Furthermore, the development of miRNA-based therapeutic delivery systems requires further research and innovation. Researchers have access to various delivery systems, yet none of them are perfect. Each of these mentioned vectors has undeniable advantages, forming the basis for the creation of hybrid vectors. Viral/non-viral carriers could combine high gene transfer efficiency with a good safety profile and tissue specificity. Additionally, the potential of artificial intelligence should not be overlooked, as it can enhance the process of developing new molecules by analyzing vast databases [178]. The main challenges include not only the immunogenicity of carriers but also their side effects, as well as the costs of production and modification according to research needs. It must be acknowledged that this aspect of developing new, effective, and personalized lung cancer treatments will require significant financial investments and a long period before a breakthrough is achieved.

## 5. Future Directions and Challenges

### 5.1. Advances in miRNA Research in the Context of Personalized Medicine

MicroRNAs are small, noncoding RNA molecules that have a significant impact on regulating gene expression, affecting numerous cellular functions, from proliferation and differentiation to apoptosis [181]. They are particularly relevant in cancer biology, where they act both as tumor suppressor genes and oncogenes. Emerging trends in miRNA research have highlighted their potential in precision medicine, especially in cancer therapy. miRNAs are promising in shaping how cancer treatments are processed and function within the body, directly impacting their effectiveness and the risk of side effects. Genetic variations, such as single nucleotide polymorphisms (SNPs) located in miRNA binding regions, can interfere with the interaction between miRNAs and their target mRNAs. This disruption may alter an individual’s susceptibility to cancer or influence the course of the disease. These findings point to the importance of designing treatment plans that are tailored to each patient’s unique miRNA profile [182,183].

The combination of miRNA and chemotherapeutic agents is a promising strategy to overcome chemotherapy resistance in cancer treatment. However, it is difficult to obtain clinical trial data on such combinations. Detailed pharmacokinetics and pharmacodynamics data are needed to fully understand their effects [184]. The growing interest in cancer biomarkers has led to the discovery of changes in miRNA expression and polymorphisms in miRNA genes, which are associated with responses to anticancer treatments [185]. Various miRNAs have been identified as predictors of sensitivity to anticancer treatments. Specifically, miR-30c, miR-130a, and miR-335 are found to be downregulated in several chemoresistant cell lines. Additionally, restoring miR-34 in p53-deficient human gastric cancer cells has been shown to induce chemosensitization [186]. Elevated serum levels of miR-21 have been observed in patients who are resistant to chemotherapy in ovarian, pancreatic, and breast cancers [185,187]. Variations in miRNA expression levels among individuals can affect how they respond to drugs, influencing both the effectiveness and toxicity of the treatments [185].

Note that a growing body of evidence is showing that miRNAs might confer benefits in the detection or prediction of drug toxicity for improving the safety of drugs, which is one of the biggest problems facing the pharmaceutical industry, clinical medicine, and public health. Consequently, miRNA variability could serve as early non-invasive biomarkers, with their levels potentially integrated into drug safety evaluations. The use of miRNAs as biomarkers for drug-induced toxicity offers the advantage of identifying the specific target organ affected by adverse effects due to their high tissue specificity, as well as pinpointing the precise toxic agent since different drugs can alter the expression of different miRNAs [185].

### 5.2. Challenges in Translating miRNA-Based Diagnostics and Therapies into Clinical Practice

Despite the significant potential of miRNAs in personalized medicine, a number of challenges hinder their clinical translation. The major challenge involves the fact that the interactions of miRNA with mRNA are very specific and complex; one miRNA can target multiple mRNAs, while one mRNA may be regulated by multiple miRNAs, developing a complex regulatory network. Lack of specificity might cause off-target effects that further complicate the development of miRNA-based therapies [170,188]. Therefore, miRNA-based therapy has the potential to trigger entire cascades of previously unknown and unavoidable consequences. The complexity of interactions can result in unpredictable adverse events that may pose a risk to the patient. A good example illustrating this problem is the anti-miR-122 drug RG-101. This substance seemed quite promising in Phase I clinical trials, but the study was stopped in Phase II due to unexpected cases of hyperbilirubinemia [189]. Furthermore, there are methodological issues related to the measurement of circulating miRNAs—such as variability in sample collection, processing, and analysis of their reliability as biomarkers [183]. Clinical trials of miRNA-based therapies have also been hindered by expression variability and limited clinical efficacy [170]. In addition, one of the major hurdles in miRNA-based therapeutics is the delivery system. There is an urgent need for the development of novel in vivo delivery methods to selectively target cells or tissues. Moreover, localized delivery of miRNA therapies remains a major challenge that requires further efforts to resolve [190]. Targeted delivery methods that could be applied in this field include lipid and polymer nanoparticles, packaging in cellular or extracellular vesicles, hybrid systems, and viral vectors [191]. It should also be noted that systemic delivery of miRNAs, similar to other nucleic acids, can trigger the innate immune system, causing toxicities and unwanted side effects. Administering miRNA duplexes systemically can induce the secretion of inflammatory cytokines and type I interferons through Toll-like receptors [192]. In the case of therapies using miRNA, one should take into account the possibility of unintentional activation of the patient’s immune system. MRX34, a miR-34a mimic, demonstrated therapeutic potential in preclinical models. miR-34a was found to downregulate PD-L1 expression in acute myeloid leukemia and to stimulate CD8^+^ T cell infiltration of tumor tissue in a mouse model of non-small cell lung cancer. Unfortunately, during clinical trials, 5 patients developed serious complications related to immune activation, leading to the termination of the clinical trial in Phase I [193].

Another issue that requires discussion is the ethical aspects of using miRNA-based therapies, which are moving toward clinical translation. To meet the upcoming challenges, the Food and Drug Administration (FDA) in November 2024 prepared a draft of recommendations for the assessment of the safety of oligonucleotide-based therapies. The document describes the risk evolution for the cardiovascular, respiratory, and nervous systems, which are defined as key systems. It discusses pharmacokinetics and the mechanisms by which oligonucleotides (including miRNAs) exert their therapeutic effects. In addition, it emphasizes the importance of identifying effects related to the targeted action (on-target) and off-target effects. The guidelines also cover the aspect of planning toxicological studies, including the appropriate selection of species and setting the initial doses of the substances tested for clinical trials. Additionally, it discusses the issues of assessing genotoxicity, reproductive toxicity, immunotoxicity, and carcinogenicity [194].

The importance of personalized medicine has also been noticed by the European Union, which supports the broad use of personalized medicine, with particular emphasis on its use in cancer diagnostics and therapy. It is worth noting that new types of advanced therapies bring with them legislative and ethical challenges. Insufficient knowledge of medical procedures among patients may raise justified doubts about seeking informed consent to undergo therapy. In addition, the issue of managing genetic information, which is aggregated during the diagnostic and therapeutic process, seems problematic. Therefore, there is a risk of genetic discrimination against certain groups of patients, which should force organizations and countries to implement measures that will prevent such events [195].

### 5.3. Emerging Trends in miRNA Research: Multi-Omics Approaches and Integration with Other Biomarkers

The multi-omics approach combines various “omics” techniques such as genomics, transcriptomics, proteomics, and metabolomics to gain a comprehensive understanding of biological systems [196]. Recent years have seen the development of high-throughput omics technologies, and integrative approaches have become necessary for biomedical research to take full advantage of these data. Machine learning algorithms integrate genetic, proteomic, and metabolomic data to facilitate the discovery of complex biological systems. These algorithms enable the identification of new biomarkers that are important for the precise prediction of diseases, the classification of patients, and the realization of precision medicine. Machine learning opens completely new avenues for the integration and analysis of diverse omics data in the further investigation of systems biology [197]. It is cumbersome to handle and interpret large datasets of RNA manually. Biological research has changed with the introduction of artificial intelligence, efficiently extracting insights from complex data. Machine learning algorithms, especially deep learning methods, are considered efficient in the identification of key miRNAs in different cancers and the development of prognostic models. The integration of AI has resulted in the establishment of huge databases of miRNAs in the identification of mRNA and gene targets, increasing understanding and applications in cancer research [198]. For example, the development of miRBind, a deep learning-based tool, addresses the limitations of traditional miRNA target binding site prediction methods [199]. Similarly, the MTLMDA model (Multi-Task Learning Model for miRNA-Disease Associations) exemplifies the application of advanced technologies in studying miRNA/disease associations. By integrating both miRNA/disease and gene/disease networks, this model enhances prediction accuracy [200].

An interesting topic is the integration of miRNAs with other markers to achieve the best possible diagnostic outcomes. One example of such an approach is the use of miRNAs combined with DNA methylation biomarkers in sputum for the early detection of non-small cell lung cancer (NSCLC). Researchers analyzed the expression of three miRNAs (miR-21, miR-31, and miR-210) and the methylation of three genes (RASSF1A, PRDM14, and 3OST2) in sputum samples from NSCLC patients and cancer-free smokers. The combined analysis of two miRNAs (miR-31 and miR-210) and two genes (RASSF1A and 3OST2) showed higher sensitivity and specificity for detecting NSCLC compared to individual biomarker panels. The integration of these biomarkers improves the accuracy of early NSCLC detection [201].

Lastly, plant miRNAs have become a subject of interest due to their crucial role in inter-kingdom gene regulation and their potential therapeutic usage in the treatment of disorders. If plant-derived miRNAs can be developed into phyto-drugs, they could provide benefits such as lower toxicity, precise targeting, and effective disease regulation. Although miRNA and plant-based miRNA therapeutics remain underexplored, they hold significant promise for advancing disease diagnosis and treatment in the future [202].

### 5.4. Conflicting miRNA Expression Patterns in Lung Cancer Subtypes

One of the most crucial challenges is diverse miRNA expression patterns in different lung cancer subtypes. One of the earliest studies suggesting a potential prognostic role for miR-21 in NSCLC was conducted by Markou et al., who observed elevated miR-21 expression in tumor tissues compared to matched standard samples [203]. Later, miR-21 was proposed as a critical post-transcriptional regulator with potential use as a tumor-specific biomarker for cancer diagnosis, prognosis, and monitoring treatment response—particularly in lung cancer, where it has been widely studied [204]. Wang et al. further demonstrated that miR-21 is consistently overexpressed in lung cancer tissues and facilitates tumor progression and cell migration by suppressing negative regulators of the RAS/MEK/ERK and MAPK/ERK signaling pathways, as well as the expression of KIBRA [205]. Together, these findings highlight the potential of miR-21 as a biomarker for lung cancer [206,207]. However, Jenike and Halushka indicated that, unfortunately, miR-21 cannot be regarded as a disease-specific biomarker if it is associated with numerous conditions. Although its expression levels may fluctuate in bodily fluids during various disease states, these changes lack diagnostic specificity. Therefore, they recommended that researchers in the miRNA field focus on other miRNAs with similarly broad associations when designing biomarker studies [208]. 

The let-7 miRNA family, known for its tumor-suppressive role, is generally downregulated in LUAD and LUSC, but differences in regulatory networks have been observed. In LUAD, let-7 targets KRAS and MYC, and its downregulation is associated with poor prognosis and increased tumor growth [209]. In contrast, in LUSC, let-7 downregulation also occurs, but downstream targets such as HMGA2 and cyclin D2 play a more dominant role in the pathogenesis [210]. This indicates that although let-7 expression is decreased in both subtypes, the functional consequences and therapeutic implications may differ.

miR-34a, a direct transcriptional target of p53, is another miRNA with subtype-specific effects. It is consistently downregulated in LUAD and has been shown to inhibit proliferation and induce apoptosis by suppressing CDK6 and BCL2 [211]. However, in LUSC, its expression levels show greater heterogeneity, and some studies report no significant correlation with patient survival or therapy response. For example, Raponi et al. found that miR-34a expression in LUSC did not reliably predict overall survival and was not significantly different from adjacent normal tissue in a subset of patients [212].

These inconsistencies emphasize the need to stratify patient samples by histological subtype and to adopt standardized detection methodologies, such as uniform RNA extraction protocols and normalization strategies. Without these controls, variability in sample processing and patient population can lead to misleading conclusions about miRNA expression and its clinical significance.

## 6. Conclusions

microRNAs show significant promise as biomarkers for diagnosing, prognosticating, and treating lung cancer, particularly in NSCLC and SCLC. They offer non-invasive early detection with high sensitivity and specificity, especially when combined with protein markers. Tumor-suppressing miRNAs like miR-34a, let-7, and miR-29 inhibit cancer progression, while oncogenic miRNAs like miR-21 can be targeted for inhibition.

Despite their promise, challenges remain, including delivery issues, off-target effects, and technical difficulties in measuring circulating miRNAs. Integrating miRNAs with chemotherapy could address resistance, and their role in personalized medicine is evolving to predict drug efficacy and toxicity. Combining miRNAs with other biomarkers, such as DNA methylation, enhances early detection.

For successful clinical application, improving delivery systems, measurement reliability, and overcoming immune responses are crucial. Plant-based miRNAs also present an exciting research opportunity. With advancements in multi-omics and AI, miRNAs could revolutionize precision medicine and improve patient outcomes.

## Figures and Tables

**Table 2:** Detailed mechanisms and targets of individual tumor-suppressor miRNAs.

Tumor Suppressor miRNA	Targets/Regulators	Mechanism	Ref.
**Apoptosis**			
miR-7	*PSME3*, *BCL-2*, *Pax6*, *PTK2*, *CCND1*, FAK	Mir-7 downregulates *PSME3*, leading to reduced cell proliferation; it targets *BCL-2*, promoting apoptosis by reducing anti-apoptotic signals; it inhibits Pax6, which downregulates the ERK/MAPK signaling pathway, and *PTK2* (FAK), which regulates cell migration and survival; it also downregulates *CCND1* to arrest the cell cycle and reduce proliferation.	[31,32]
miR-34b-3p	*CDK4*	Acts as a tumor suppressor in NSCLC by targeting the 3′-UTR of CDK4 mRNA, leading to decreased CDK4 expression, which inhibits cell proliferation and induces cell cycle arrest and apoptosis.	[30]
**Apoptosis, cell cycle arrest**			
mir-143	*PSME3*, *BCL-2*, *Pax6*, *PTK2 (FAK)*, *CCND1*	miR-143 downregulates *PSME3* to reduce cell proliferation; targets *BCL-2* to promote apoptosis by reducing anti-apoptotic signals; inhibits *Pax6*, suppressing the ERK/MAPK pathway; downregulates *PTK2* (FAK) to limit migration and survival; targets *CCND1* to arrest the cell cycle and reduce proliferation.	[33]
**Apoptosis, metastasis**			
miR-29c	DNA methyltransferases (*DNMT3A*, *DNMT3B*) *MMPs*, VEGF, pro-apoptotic genes (e.g., Bax, Bak), Bcl-2, Mcl-1, Cyclins, CDKs	Regulates DNA methylation by targeting *DNMT3A* and *DNMT3B*, helping maintain proper epigenetic regulation, and preventing tumor suppressor gene silencing. Inhibits metastasis by targeting genes involved in extracellular matrix remodeling, such as *MMPs*, reducing tumor cell migration and invasion. Promotes apoptosis by regulating pro-apoptotic proteins like Bax and Bak, Downregulates anti-apoptotic proteins (Bcl-2, Mcl-1), inducing apoptosis and sensitizing cells to radiation-induced cell death.	[34]
miR-134	*FOXM1*, *CCND1*, *EGFR*, *ITGB1*, *Cyclin D1*, *Cyclin D2*, *CDK4*, *MMP-7*, *MMP-9*, *Caspase-3*, *Bcl-2*	Suppresses EMT, migration, and invasion by targeting *FOXM1* and *CCND1*; reduces *EGFR* expression, inhibiting cell growth and proliferation; promotes apoptosis via Caspase-3 and Bcl-2 regulation; reduces *MMP-7* and *MMP-9* for anti-migration effects.	[35,36]
**Angiogenesis, metastasis**			
miR-29	*LOXL2*, *Wnt-1*, *DNMT3A*, *DNMT1*	Suppresses cancer progression by targeting *LOXL2* (oncogene) and by demethylating Wnt-1. It also targets DNMTs to reduce hypermethylation of tumor suppressor genes.	[37]
**Antiproliferation, metastasis**			
miR-203	*RGS17*	miR-203 targets the 3′-UTR of RGS17 mRNA, leading to post-transcriptional downregulation of RGS17 expression. The downregulation of RGS17 inhibits cell proliferation, migration, and invasion in NSCLC cells. RGS17 contains a palmitoylation site that regulates its subcellular localization and G-protein receptor selectivity, promoting tumor progression. Overexpression of RGS17 reverses the effects of miR-203.	[38]
**Cell cycle arrest**			
miR-195	*CHECK1*	Downregulation of CHEK1 expression and delays cell cycle progression.	[39]
**Drug resistance**			
miR-218	*SLIT2/3*, IL-6R, *JAK3*, *STAT3* (phosphorylated)	It regulates the expression of its host gene *SLIT2/3* and targets IL-6R, *JAK3*, and phosphorylated *STAT3*. Overexpression of miR-218 inhibits cell survival, migration, and invasion. miR-218 expression also influences the *ALDH1A1*-positive lung cancer cell population, affecting their tumorigenic potential.	[40]
miR-199a-3p, miR-199a-5p	*Rheb, MAP3K11*	Regulates mTOR signaling pathway, inhibits cell proliferation, and migration, and promotes apoptosis; regulatory axis with *MAP3K11* in NSCLC.	[41]
**EMT, metastasis**			
miR-138	*SIRT1*, *ZEB2*, *EGFR*, *EZH2*, *GPR124*, *YAP1*	miR-138 is downregulated by TGFβ1 exposure, leading to increased stemness via EMT. It targets *SIRT1* to reduce AMPK signaling, *ZEB2* to suppress EMT, and *EGFR/EZH2* to inhibit proliferation. MiR-138 also targets GPR124 to restore EGFR TKI sensitivity in NSCLC. Additionally, lncRNAs like PFAR and SNHG12 regulate miR-138 expression, impacting lung fibrosis and cancer progression.	[42]
**Metastasis**			
miR-15a	*ACSS2*, fatty acid synthesis	Acts as a tumor suppressor by inhibiting the proliferation, migration, and invasion of lung cancer cells. It targets the 3′-UTR region of *ACSS2*, reducing acetate uptake and acetyl-CoA activity, leading to decreased histone H4 acetylation. This disrupts fatty acid synthesis and lipid metabolism, impairing metastasis. Under hypoxic conditions, miR-15a-5p is transported into the nucleus, further inhibiting *ACSS2* and reducing acetyl-CoA activity and histone acetylation, thus suppressing cancer progression.	[25]
miR-16	*MEK1* (MAPK kinase 1)	miR-16 downregulates *MEK1* expression, inhibiting the ERK/MAPK signaling pathway and suppressing lung cancer cell proliferation, migration, and invasion.	[43]
miR-148	*Wnt1*, *ROCK1*, *CEA*	Suppresses cancer progression by targeting *Wnt1* and *ROCK1*, reducing cell invasion and migration, and reversing EMT in NSCLC. It also inhibits CEA in cancer cells. Overexpression of miR-148a-3p or *MAP3K9* silencing inhibits tumor growth and metastasis in vitro and in vivo.	[44,45]

**Table 4:** AUC, sensitivity, and specificity for miRNAs panels associated with lung cancer.

miRNA Panel	AUC	Sensitivity	Specificity	Ref.
miRNAs-25, 223, 141, 155, 1254	-	-	-	-
miRNAs-125a-5p, 25, 126	0.936	87.5%	87.5%	[76]
miRNAs-126, 145, 210, 205-5p	-	91.5%	96.2%	[77]
miR-143, let-7g, miR-126, let-7a, miR-145	0.90–0.93	75–85%	75–85%	[78]
miRNAs-7a-5p, 375, 1-3p, 1291, 214-3p	-	-	-	-
34-miRNA model	0.89 (Stage I); 0.88 (Stage II–IV)	59% (Stage I); 92% (Stage II–IV)	90% (Stage I); 90% (Stage II–IV)	[79]
miRNAs-155, 197, 182	0.92, 0.84 0.98	miRNA-182 100%	miRNA-182 86.5%	[80]
miRNAs-21-5p, 181-5p, 155-5p	-	-	-	-
miRNAs-199a−3p, chr17_10932, 148a−3p, 210−3p, chr1_1402, 378d, 138−5p	-	-	-	-
miRNAs-210-3p, 301a-5p	-	-	-	-
miRNAs-324-3p, 1285	0.89	85.4%	81.8%	[81]
miRNAs-320a-3p, 210-3p, 92a-3p, 21-5p, 140-3p + 4MP	-	19.1%	95%	[82]
miRNAs-6777-5p, 6780a-5p, 877-5p	0.98	88%	100%	[83]
miRNA-150, miRNA-886-3p	-	-	-	-
miRNAs-143, 100, 101-1, 101-2, 182, 183, 205, 21, 30a, 30d	-	-	-	-
miRNAs-155-5p, 223-3p, 126-3p	-	-	-	-
miRNAs-20a-5p, 152-3p, 199a-5p	-	-	-	-
miRNAs-25, 145, 210	-	-	-	-
miRNAs-375, 200c, 30c	-	-	-	-
miRNAs-101-2, 139, 182, 183, 190, 326, 944	-	-	-	-
miRNAs-142-3p, 29b	-	-	-	-
miRNAs-92a-2, 147, 574-5p	-	-	-	-
miRNAs-105-5p, 767-5p	-	-	-	-
miRNAs-22, 24, 34a	-	-	-	-

**Table 8:** A summary of the advantages and disadvantages of specific delivery systems for miRNA-based therapies.

Delivery System	Advantages	Disadvantages
**Viral vectors**		
Adenoviruses	High delivery efficiency; well researched.	Significant immunogenicity; immune response limits therapy effectiveness and repeated use.
Adeno-associated viruses	Lower immunogenic potential.	Still triggers an immune response in some cases.
Retroviruses and lentiviruses	Stable gene expression through integration into the host genome; long-term therapy potential.	Risk of insertional mutagenesis and carcinogenesis.
**Non**-**viral vectors**		
Lipid-based vectors	Low immunogenicity; high availability and variety.	Low delivery efficiency; low tissue specificity; off-target effects; susceptible to phagocytosis.
Polymeric vectors	Low immunogenicity; flexibility for modification and synthesis.	Cytotoxicity of common polymers.
**Extracellular vesicles**		
Exosomes	Low immunogenicity and cytotoxicity; high delivery efficiency.	High production costs.
Microvesicles and apoptotic bodies	Low immunogenicity and cytotoxicity.	Limited research.

**Table 9:** Current clinical trials of miRNA-based therapies.

Study Type	miRNA	Current Status of the Trial	Key Information and Conclusions	Ref.
Interventional (MRX34)	miR-34	Terminated	This trial ended early due to immune-related serious adverse events, including the death of four patients. Further studies to understand systemic immune activation and discover new delivery systems are crucial.	[179]
Interventional (MesomiR-1)	miR-16	Completed Phase I	The study showed promising results in patients with mesothelioma, although more data and research are needed to determine the effectiveness of NSCLC.	[180]
Observational	miR-21, miR-155	Completed	Current research indicates potential and the need for further studies on the application of these molecules.	[170]
